# 

**Published:** 1907-09-15

**Authors:** 


NELVILLE S. HOFF, Editor,
Ann Arbor, Mich.

Vol. LXI — SEPTEMBER, 1907 — No. 9

SAMUEL A. CROCKER & CO., Publishers
OHIO DENTAL AND SURGICAL DEPOT
35 W. FIFTH STREET, CINCINNATI, O.

PRICE, ONE DOLLAR A YEAR

Entered at the Postoffice at Cincinnati, O., as second-class mail matter

